# A New Stonefly Species, *Rhopalopsole tricuspis* (Leuctridae: Plecoptera), and Three New Records of Stoneflies from the Qinling Mountains of Shaanxi, China

**DOI:** 10.1673/031.012.4701

**Published:** 2012-03-30

**Authors:** Yu-Han Qian, Yu-Zhou Du

**Affiliations:** Institute of Applied Entomology, Yangzhou University, Yangzhou, Jiangsu 225009, China

**Keywords:** new species, redescription

## Abstract

Plecopteran species (Leuctridae) were collected from the Qinling Mountains in southern Shaanxi Province, China. This mountain range is home to nine species of Leuctridae belonging to two genera, and the species identified in this work include one new species and three new records for the Qinling Mountains, all belonging to the genus *Rhopalopsole*. The new species is named *R. tricuspis* Qian and Du, sp. nov. A redescription of *R. basinigra* Yang and Yang 1995 is supplemented. A key is provided for the adult males of Leuctridae from the Qinling Mountains.

## Introduction

Leuctridae is one of the families of Plecoptera distributed in the Nearctic, Palearctic, and Oriental Regions, established by Klapalek in 1905. Due to their small size and general absence from light traps, the number of known Chinese leuctrids is far less than in other regions. Thus far, eight leuctrid species have been recorded from China by early workers such as Klapalek (1912), Chu (1928), Wu (1935, 1973, 1949), Claassen (1940), Zwick (1973 and 1977), and Nelson and Hanson (1973). More recent efforts have been made primarily by Yang and Yang (1991, 1994, 1995), Yang et al. (2004, 2006, 2009), Du and Sivec (2005), Sivec et al. (2008), Li et al. (2010, 2011), and Qian and Du (2011).

Specimens in this study were collected from the Qinling Mountains, located in southern Shaanxi Province, China. The Qinling Mountains are an ancient fold-fault; the land began rising as early as 400 million years ago and formed the present landscape pattern about 80 million years ago (Wang 2005). It has an average elevation of 2000 to 3000 meters, with its highest mountain, Mt. Taibai, reaching an elevation of 3767 meters (Ying 1994). The Qinling Mountains include five national nature reserves and the Crested Ibis Protection Observation Station, with a total area of 2180 km^2^ (Zhang and Li 1997).

The mountains not only form the watershed between the Yangzi River and the Yellow River and create a natural boundary between the north and south of China—they also act as a boundary between the temperate and subtropical zones (Zhang et al. 1979). The Qinling Mountains are also an abundant biodiversity region, and were deemed to be a boundary between the Oriental and Palearctic Regions in China (Zhang et al. 1999). Thus, it is a key area to study Chinese insect fauna. In the present paper, one new species of *Rhopalopsole* is described, and revisions are made to some known species of leuctrids in the area. The materials studied are deposited in the Insect Collection of Yangzhou University, Jiangsu, and all specimens are preserved in 75% ethanol.

**A key to adult male species of Leuctridae from the Qinling Mountains**
1. Anal field of hind-wing large. Presternum of prothorax partially separated from basisternum (*Paraleuctra*). Tergum 10 with posterior notch, cerci with a small bulge on dorsal arm *Paraleuctra orientalis* (Chu)
Anal field of hind-wing very small. Presternum of prothorax completely separated from basisternum (*Rhopalopsole*) 2
2. Posterolateral processes modified into a chitinized bifurcated process of tergum10 *Rhopalopsole furcata* Yang and Yang
Posterolateral processes without a chitinized bifurcated process 3
3. Posterolateral processes very long, reaching just beyond the mid-line of tergum 10, crossing over the tip of the corresponding process from the other side *R. basinigra* Yang and Yang
Posterlateral processes short, not reach the mid-line of tergum 10 4
4. Hind margin of tergum 9 with two narrow bands sclerotized connected in middle and expanded on each side slightly. Subanal lobes long and with distinct ventral flaps *R. emeishan* Sivec and Harper
Hind margin of tergum 9 with only one or no sclerotized speckle. Subanal lobes flat and large 5
5. Mid-posterior margin of tergum 9 with only one sclerotized speckle 6
Hind-posterior margin of tergum 9 without sclerotized speckle 7
6. Two small spines jutting out in ventral view of tergum 9. Epiproct with a trilobed tip in dorsal view *R. tricuspis*, sp. nov.
No spines and small ridges jutting out in ventral view of tergum 9. Epiproct with a rounded tip in dorsal view *R. jialingensis* Sivec and Harper
7. Epiproct thick, terminating flattened in dorsal view *R. qinlinga* Sivec and Harper
Epiproct thin, terminating tapering in dorsal view 8
8. Tergum 10 with central sclerotized plate covered by the extended tergum 9. Lateral processes with a very short sclerotized point in dorsal view *R. shaanxiensis* Yang and Yang
Tergum 10 with central sclerotized plate separated into three small parts. Lateral processes with a long and thin point in dorsal view *R. horvati* Sivec and Harper

**Genus *Paraleuctra* Hanson, 1941 *Paraleuctra orientalis* (Chu), 1928**
*Leuctra orientalis* Chu, 1928, China J. 9: 87.
  *Leuctra orientalis* Chu: Wu, 1935, Cat. Ins., I: 131; 1938, Plecopt. Sin. 161: 1939–1940, Peking Nat. Hist. Bull., 14(2): 153; 1949, Peking Nat. Hist. Bull. 17(4): 251.
  *Leuctra orientalis* Chu: Claassen, 1940, Mem. Agr. Exp. Sta. Cornell Univ., 232: 84.
  *Rhopalopsole orientalis* (Chu): Illies, 1966, Das Tierreich 82: 118.
  *Paraleuctra orientalis* (Chu): Wu, 1973, Acta Entomologica Sinica 16(2): 98.
  *Paraleuctra orientalis* (Chu): Zwick, 1973, Das Tierreich 94: 410.
  *Paraleuctra orientalis* (Chu): Du & Sivec, 2005, In Yang X. K. (Ed.). Insect Fauna of Middle-west Qinling Range and South Montains of Gansu Province, 40.
  *Paraleuctra orientalis* (Chu): Li, Wang & Yang, 2010, Zootaxa 2350: 47.
**Material examined.** 1 ♂, **China:** Shaanxi Province, Qinling Mountain Range, Mt. Tiantai, Source of Jialing River, 1750 m, 10 June 1998, Leg. Du Yu-Zhou. 1 ♀, **China:** Shaanxi Province, Qinling Mountain Range, Zhouzhi County, Houzhenzi, Hougou, 1300 m, 26 May 1995, Leg. Du Yu-Zhou. 4 ♀♀, **China:** Shaanxi Province, Qinling Mountain Range, Railway Station of Qinling, 16 May 1995, Leg. Du Yu-Zhou.
**Remarks.** *Paraleuctra orientalis* (Chu) 1928 was redescribed by Li et al. (2010). Our examination of all the above material confirms that the male and female resembles *P. cercia* (Okamoto 1922). They have deeply forked cerci with long ventral arms; paraproct without an expansion, male subgenital plate with a deeply excavated hind margin and the female subgenital plate is strongly sclerotized and divided in the middle of the hind margin. We also have found a small projection on the ventral arm of the cerci.

**Figures 1–5.**
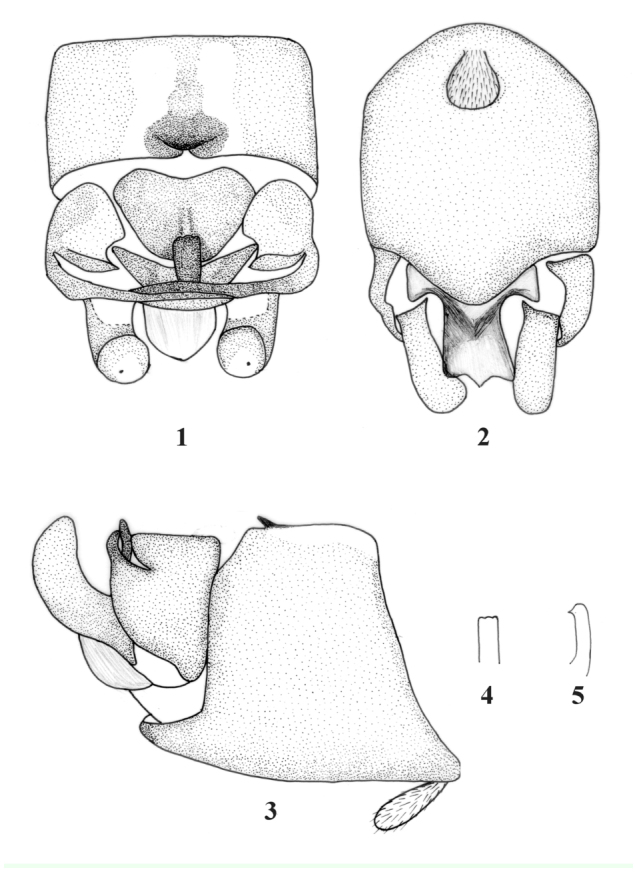
*Rhopalopsole basinigra* male structures. (1) Terminal abdominal segments of male, dorsal view; (2) Terminal abdominal segments of male, ventral view; (3) Terminal abdominal segments of male, lateral view; (4) Epiproct, dorsal view; (5) Epiproct, lateral view. High quality figures are available online.

**Genus *Rhopalopsole* Klapálek, 1912**
***Rhopalopsole basinigra*** Yang and Yang, 1995 (new record) (Figures 1–5)
  *Rhopalopsole basinigra* Yang and Yang, 1995, Plecoptera: Leuctridae. In: Insects and Macrofungi of Gutianshan, Zhejiang, 20.
  *Rhopalopsole basinigra* Yang and Yang, 1995, Plecoptera: Perlidae. In: Insects of Baishanzu Mountain, Eastern China, 61.
  *Rhopalopsole basinigra* Yang and Yang: Harrison and Stark, 2008, Illiesia 4(7): 79.
**Material examined.** 3 ♂♂, **China:** Shaanxi Province, Qinling Mountain Range, Mt. Tiantai, Source of Jialing River, 1800 m, 10 June 1998, Leg. Du Yu-Zhou. 23 ♂♂, **China:** Shaanxi Province, Qinling Mountain Range, Zhouzhi County, Hougou, Houzhenzi, 500 m, 24–26 May 1995, Leg. Du Yu-Zhou. 1 ♂, **China:** Shaanxi Province, Qinling Mountain Range, Railway Station of Qinling, 15 May 1995, Leg. Du Yu-Zhou.
**Adult habitus.** Head dark brown, wider than prothorax, three ocelli, hind ocelli much closer to the eyes than to each other, antennae yellowish brown, palpi light brown. Prothorax light brown, quadrate, longer than wide, angles rounded and some brown stripes on it. Legs light brown. Wings hyaline, veins light brown.
**Male.** Body length 6.0 mm, length of forewing 5 mm. Ventral lamella on tergum 9 rounded (Figure 2), a small fairly sclerotized semicircular ridge jutting out before the mid-posterior margin, with mid-posterior margin indented (Figure 1). Tergum 10 with a central sclerotized plate, two sclerotized stripes stretching downward to the posterior margin in the middle of the central plate; the posterior margin of plate more sclerotized, transverse sclerites triangular-semicircular with rounded angles (Figure 1). Posterolateral processes very long and narrow, reaching just beyond the mid-line of segment 10, thus crossing over the tip of the corresponding process from the other side (Figures 1 and 3). Epiproct stocky and upper curved, apex flattened, in dorsal view with a trilobed tip with rounded angles (Figures 1 and 4), a spine in the middle lobe located at a short distance to the tip (Figure 5).
**Remarks.** Yang (1995) did not describe in detail *R. basinigra*. We checked the types of *R. basinigra* in the Insect Collection of Beijing Agricultural University. It has a ridge that juts out before the mid-posterior margin of tergum 9; with long lateral processes reaching beyond the mid-line of tergum 10; apex of epiproct flattened, with a trilobed tip and a downcast spine in the middle lobe at a short distance to the tip. Epiproct of some species like in Figure 4 having one parallel-sided, though Figure 1 does not—they may have variations.

***Rhopalopsole emeishan* Sivec and Harper, 2008 (new record)**
*Rhopalopsole emeishan* Sivec and Harper, 2008, Sivec et al., Scopolia 64: 16.
**Material examined.** 14 ♂♂, **China:** Shaanxi Province, Qinling Mountain Range, Huoditang, 5 Jun. 1998, Leg. John C. Morse and Du Yu-Zhou. 11 ♂♂, **China:** Shaanxi Province, Qinling Mountain Range, Mt. Tiantai, Source of Jialing River, 1800 m, 10 June 1998, Leg. Du Yu-Zhou.
**Remarks.** Recently described species from Sichuan Province, China. Epiproct curved hook-like, its tip narrowing and turned forward. Distal ends of cerci with a little macula.

***Rhopalopsole furcata* Yang and Yang, 1994**
*Rhopalopsole furcata* Yang and Yang, 1994, Entomotaxonomia 16(3): 190.
*Rhopalopsole furcata* Yang and Yang: Du and Sivec, 2005, In: Insect Fauna of Middle-west Qinling Range and South Montains of Gansu Province, 40.
*Rhopalopsole furcata* Yang and Yang: Harrison and Stark, 2008, Illiesia, 4(7): 79.
**Material examined.** 10 ♂♂, **China:** Shaanxi Province, Qinling Mountain Range, Mt. Tiantai, Source of Jialing River, 1800 m, 9 June 1998, Leg. Du Yu-Zhou. 2 ♂♂, **China:** Shaanxi Province, Qinling Mountain Range, Zhouzhi County, Angou, Houzhenzi, 1250 m, 2 June 1998, Leg. Du Yu-Zhou.
**Remarks.** We checked the types of *R. furcata* and *R. sinensis* Yang and Yang 1995, deposited in the Insect Collection of China Agricultural University, *R. furcospina* (Wu) 1973 in the Institute of Zoology Chinese Academy of Science. We consider that the three form a species complex. Yang (1995) distinguished *R. sinensis* and *R. furcata* by the body color; but this is not accurate, because the type was preserved in 75% alcohol, and body color will fade over time. The difference between *R. furcata* and *R. sinensis* is the presence of a sclerotized speckle on the midposterior margin on tergum 9. *Rhopalopsole sinensis* has a sclerotized speckle, but *R. furcata* lacks it. In *R. furcospina*, the central plate of tergum 10 has two lateral sclerites. But *R. furcata* lacks the two lateral sclerites; the two lateral sclerites are fused with the central plate in *R. sinensis*.

***Rhopalopsole horvati* Sivec and Harper, 2008 (new record)**
*Rhopalopsole horvati* Sivec and Harper, 2008, Sivec et al., Scopolia 64: 75.
**Material examined.** 2 ♂♂, **China:** Shaanxi Province, Qinling Mountain Range, Liuba County, Miaotaizi (Zhangliang Temple), 1400 m, 8 June 1998, Leg. Du Yu-Zhou.
**Remarks.** Recently described species from Sichuan Province, China.

***Rhopalopsole jialingensis* Sivec and Harper, 2008**
*Rhopalopsole jialingensis* Sivec and Harper, 2008, Sivec et al., Scopolia 64: 83.
**Material examined.** 39 ♂♂, **China:** Shaanxi Province, Qinling Mountain Range, Mt. Tiantai, Source of Jialing River, 1750–1800 m, 8–10 June 1998, Leg. Du Yu-Zhou. 4 ♂♂, **China:** Shaanxi Province, Qinling Mountain Range, Zhouzhi County, Houzhenzi, Hougou, 1300 m, 3 June 1995, Leg. Du Yu-Zhou.
**Remarks.** Recently described species from Shaanxi Province, China.

***Rhopalopsole qinlinga* Sivec and Harper, 2008**
*Rhopalopsole qinlinga* Sivec and Harper, 2008, Sivec *et al.*, Scopolia 64: 77.
**Material examined.** 3 ♂♂, **China:** Shaanxi Province, Qinling Mountain Range, Liuba County, Miaotaizi (Zhangliang Temple), 1400 m, 8 June 1998, Leg. Du Yu-Zhou.
**Remarks.** Recently described species from Shaanxi Province, China.

***Rhopalopsole shaanxiensis* Yang and Yang, 1994**
*Rhopalopsole shaanxiensis* Yang and Yang, 1994, Entomotaxonomia 16(3): 189.
**Material examined.** 11 ♂♂, **China:** Shaanxi Province, Qinling Mountain Range, Huoditang, 5 June 1998, Leg. Du Yu-Zhou. 3 ♂♂ , same data, Leg. John C. Morse. 6 ♂♂, same data, Leg. Sun Chang-Hai and Yang Lian-Fang. 1 ♂, same data, Leg. Ma Yun.
**Remarks.** Recently described species from Shaanxi Province, China.

**Figures 6–9.**
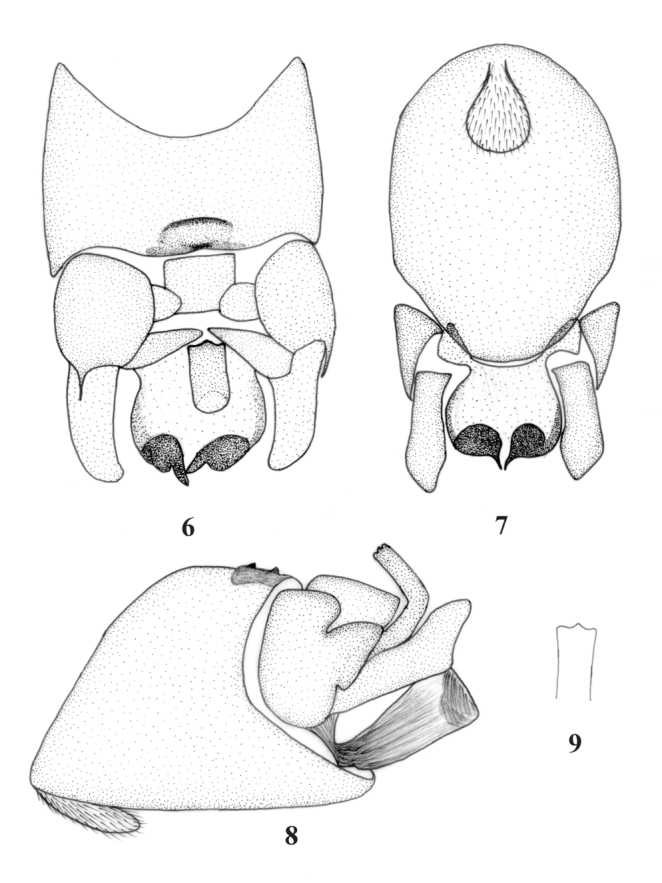
*Rhopalopsole tricuspis* sp. nov. male structures. (6) Terminal abdominal segments of male, dorsal view; (7) Terminal abdominal segments of male, ventral view; (8) Terminal abdominal segments of male, lateral view; (9) Epiproct, dorsal view. High quality figures are available online.

***Rhopalopsole tricuspis* Qian and Du, sp. nov. Figures 6–9)**
**Material examined.** Holotype ♂, **China:** Shaanxi Province, Qinling Mountain Range, Liuba County, Miaotaizi (Zhangliang Temple), 1400 m, 8 June 1998, Leg. Du YuZhou. Paratype 5 ♂♂, same data as holotype. All type material deposited in the Insect Collection of Yangzhou University, Jiangsu, China.
**Adult habitus.** Head brown, wider than prothorax, three ocelli and hind ocelli much closer to the eyes than to each other, antennae and palpi light brown. Prothorax light brown, subquadrate, angles rounded and some faintly brown stripes on it. Legs light brown. Wings hyaline, veins light brown.
**Male.** Body length 5.0 mm, forewing length 5.5 mm. On tergum 9, the ventral lamella somewhat large and rounded (Figure 7), a sclerotized speckle before the mid-posterior margin, slightly indents at mid-posterior in dorsal view (Figure 6), two small spines jut out in ventral view (Figure 8). Tergum 10 with a small central plate and two lateral bulging lobes on each side of central plate, transverse sclerites triangular with rounded angles (Figure 6). Posterolateral processes extending upwards and triangular-like in lateral view (Figure 8). Epiproct thick, elongate and upper curved, terminating in a flattened, trilobed tip with rounded angles in dorsal view (Figures 6 and 9), an L-like projection and upper curved in lateral view (Figure 8). Subanal lobe upturned in lateral aspect (Figure 8) separated into a basal ventral lobe and two upper distal lobes, upper distal lobes more sclerotized and tip sharp (Figures 6 and 7). Cerci short and distinctly upturned in lateral aspect, no spine (Figure 8).
**Female.** Unknown.
**Etymology.** The species name is derived from the shape of the epiproct in dorsal view.
**Remarks.** This new species seems similar to *R. shaanxiensis* group (Sivec et al. 2008). It seems similar to *R. qinlinga* Sivec and Harper 2008 in having two lateral bulging lobes on each side of central plate, epiproct thick and flattened in dorsal view. But it can be distinguished from *R. qinlinga* by the outline of tergum 9, epiproct distal ends and subanal lobe. In *R. qinlinga*, no ridge juts out before the mid-posterior margin of tergum 9, epiproct distal ends blunt rounded and no split, subanal lobes separated into a basal lobe and an upper distal lobes in ventral view, each with strong dark sclerotized stripes on it.
